# Chondroblastoma patella presenting as a pathological fracture

**DOI:** 10.4103/0019-5413.38592

**Published:** 2008

**Authors:** Narayan Gudi, VR Venkatesh Reddy, KJC Chidanand

**Affiliations:** Sri Devaraj Urs Medical College, Kolar, Karnataka, India

**Keywords:** Chondroblastoma patella, pathological fracture

## Abstract

A 24-year-old male presented with an inability to walk after a trivial fall. He had pain and mild swelling anterior to the right knee for the past one year. X-ray showed a transverse fracture of patella with a lytic lesion occupying most of the two halves of the patella. Fine needle aspiration cytology (FNAC) of the lytic lesion revealed a few osteoclastic giant cells and occasional osteoblasts against a hemorrhagic background. Patellectomy was performed. Histology revealed trabecular bone admixed with proliferating chondroid tissue at places admixed with myxoid and fibrous tissue with focal areas of calcification suggestive of chondroblastoma. Focal areas showed osteoclastic giant cells with areas of hemorrhage. The purpose is to present a rare tumor occurring at an unusual site which presented as pathological fracture.

## INTRODUCTION

Chondroblastoma was first described as calcified giant cell tumor by Ewing.^1^ Codman^2^ described it as epiphyseal chondromatous giant cell tumor. Jaffe and Lichtenstein^3^ named it as chondroblastoma, a rare benign cartilaginous tumor. Its incidence is only 1-3% of all primary benign bone tumors. The typical localization of a chondroblastoma is the epiphysis of long tubular bones; patella is a very unusual site. Chondroblastoma characteristically arises in the epiphyses of long bones in young adults.^4^,^5^ Occurrence of chondroblastoma in the patella is still rare with only case reports being reported in the English language literature. The association of fracture patella with chondroblastoma is still rare with best of our knowledge this is seventh such case.

## CASE REPORT

**Figure 1:**
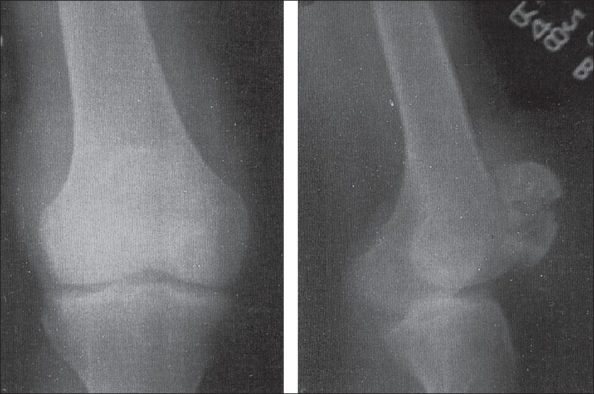
X-ray anteroposterior (AP) and lateral view of knee joint shows a transverse fracture of patella with a lytic lesion occupying most of the two halves of the patella. There was no periosteal reaction. A thin sclerotic rim was present with fine matrix calcification with in the lesion

**Figure 2:**
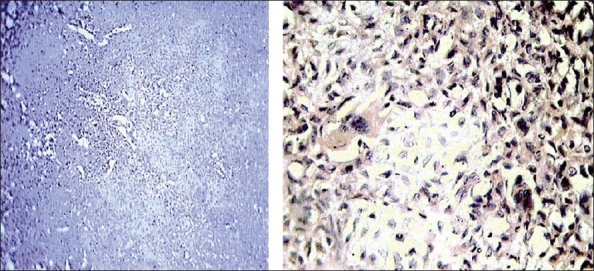
Histopathology revealed, trabecular bone admixed with proliferating chondroid tissue at places admixed with myxoid and fibrous tissue with focal areas of calcification. Focal areas show osteoclastic giant cells with areas of hemorrhage

A 24-year-old male presented with inability to walk after a trivial fall. He had pain and noticed mild swelling in the right knee for the past one year. Clinically, patient had tense swelling and tenderness over the right patella. The movements of right knee were painful and straight leg raising test was not possible. X-ray showed a transverse fracture of the patella with a lytic lesion occupying most of the two halves of the patella. A thin sclerotic rim was present with fine matrix calcification within the lesion [Figure 1]. Chest X-ray was normal. Solitary bone cysts, aneurysmal bone cyst, giant cell tumor, chondroblastoma were considered as clinicoradiological differential diagnoses. FNAC of the lytic lesion revealed a few osteoclastic giant cells and occasional osteoblasts against a hemorrhagic background. Patellectomy was performed and histopathology revealed trabecular bone admixed with proliferating chondroid tissue at places admixed with myxoid and fibrous tissue with focal areas of calcification. Focal areas showed osteoclastic giant cells with areas of hemorrhage suggestive of chondroblastoma [Figure 2]. At the end of two years the patient has full range of movements.

## DISCUSSION

Chondroblastoma is being reported as 1-3% of all primary benign bone tumors.^1^–^4^ This tumor arises from immature cartilage cells. Patella though a sesamoid bone, is formed from a cartilage focus.^3^,^4^ It most often presents in the second and third decade, almost always in the distal epiphysis of the femur, proximal humerus and proximal tibia. Occurrence of chondroblastoma in a site like the patella is very rare with an estimated occurrence of 2%.^9^ The localization and radiographic findings are similar to giant cell tumor of bone, so the tumor was categorized as an epiphyseal chondrogenic giant cell tumor by Codman^1^ and as a benign calcifying giant cell tumor by Ewing until Jaffe and Lichtenstein reported the entity of chondroblastoma in 1943.^3^

Primary patella tumors are very rare; the differential diagnosis includes benign and malignant tumors and metabolic disorders.^1^–^4^,^7^ Giant cell tumor is one of the likely differential diagnoses with tumors of the patella. There are several differences between chondroblastoma and giant cell tumor. Radiographically, chondroblastoma has clear boundaries whereas giant cell tumor has faded boundaries. Histologically chondroblastoma has calcification within the tumor, but giant cell tumor does not.^7^-^9^ The recommended treatment of chondroblastoma includes a biopsy to determine histology followed by curettage and bone grafting. Chemotherapy is not used in chondroblastoma.^1^,^7^,^8^

In our patient patellectomy was done as only minimal healthy patella was remaining. Complications of chondroblastoma include pathological fracture and rarely malignant transformation.^5^
